# Targetable alterations in invasive pleomorphic lobular carcinoma of the breast

**DOI:** 10.1186/s13058-020-01385-5

**Published:** 2021-01-13

**Authors:** Gregory M. Riedlinger, Sonali Joshi, Kim M. Hirshfield, Nicola Barnard, Shridar Ganesan

**Affiliations:** 1 Rutgers Robert Wood Johnson Medical School, New Brunswick, NJ 08901 USA; 2 Rutgers Cancer Institute of New Jersey, 195 Little Albany St Room 3553, New Brunswick, NJ USA; 3 Merck, Rahway, NJ USA

**Keywords:** Pleomorphic, Lobular, ERBB2, PIK3CA, Genomics

## Abstract

**Background:**

Invasive pleomorphic lobular carcinoma (PLC) of the breast is a subtype of invasive lobular cancer which compromises approximately 1% of all epithelial breast malignancies and is characterized by higher nuclear pleomorphism and poorer prognosis than classic invasive lobular cancer (ILC). Since PLC is more aggressive than classical ILC, we examined the underlying molecular alterations in this subtype of breast cancer to understand the possible benefit from targeted therapies.

**Methods:**

In this study, we investigate the clinical characteristics and molecular alterations in 16 PLC from our institution. Additionally, we examined the clinical and genomic features in 31 PLC from the Cancer Genome Atlas (TCGA).

**Results:**

Overall, our analysis of PLC found that 28% had activating ERBB2 mutations, 21% had ERBB2 amplification, and 49% activating PIK3CA mutations. Among cases from our institution, we found 19% with activating ERBB2 mutations, 25% had ERBB2 amplification, and 38% with activating PIK3CA mutations. In data from TCGA, 32% had activating ERBB2 mutations, 19% had ERBB2 amplification, and 55% had activating PIK3CA mutations. While classic ILC in TCGA had similar percentages of PIK3CA alterations compared to PLC, activating ERBB2 alterations were exceedingly rare, with no activating ERBB2 mutations and only one case with ERBB2 amplification. Interestingly, in further examining TCGA data which included FGFR1 and PTEN, 94% of PLC had alterations in ERBB2, FGFR1, or the PI3K pathway.

**Conclusions:**

Our results show a high frequency of ERBB2 and PIK3CA alterations in PLC and suggest all PLC should be tested for potential therapeutic targeting.

**Supplementary Information:**

The online version contains supplementary material available at 10.1186/s13058-020-01385-5.

## Background

The second most common histologic subtype of invasive breast cancer is invasive lobular carcinoma (ILC) compromising up to 15% of all cases. It is more frequently multifocal and bilateral than other primary breast cancers. In the classical form, ILC is associated with a good prognosis and is typically low grade and estrogen receptor and progesterone receptor positive [1, 2]. It is characterized by non-cohesive cancer cells invading the stroma in a single-file pattern, without mass formation or calcification making detection by physical examination or mammography more difficult. The lack of cellular cohesion is principally a result of lack of E-cadherin protein expression in ~ 90% of cases [1, 2]. This feature is often employed to diagnose lobular versus ductal lesions histologically through immunohistochemistry.

While classical ILC generally has a good prognosis, several variants of ILC have been described. Among these is pleomorphic lobular carcinoma (PLC) which was first described by Page and Anderson in 1987 but was not officially recognized by the WHO until 2003 [3, 4]. PLC has been reported to present at a more advanced stage compared to invasive ductal carcinoma (IDC) or classical ILC and be more likely to recur compared to classical ILC [5, 6]. PLC has been associated with postmenopausal status and older age and accounts for ~ 15% of ILC and < 1% of all breast cancers [7]. Similar to classical ILC, PLC histologically shows discohesive cells in a linear invasive pattern lacking E-cadherin protein expression. In contrast to classical ILC, PLC displays a high degree of cellular pleomorphism, higher mitotic index, eosinophilic cytoplasm, nuclear hyperchromasia, and prominent nucleoli [8]. Molecular characterization suggests PLC shares many of the same molecular alterations as classical ILC and is therefore best characterized as a variant, including similar expression of estrogen and progesterone receptors [9]. Additionally, PLC is reported to more frequently have ERBB2 amplification or TP53 alterations than classical ILC and more typical of high grade IDC [10, 11].

More recently, several small studies have performed sequencing based analysis to further characterize the molecular alterations in PLC and have reported frequent mutations in ERBB2 and PIK3CA [12–14]. PLC is more aggressive than classical ILC and may benefit from targeted therapies. To gain a better of the underlying molecular alterations in PLC and the potential for therapeutic intervention, we report here on the characterization of 47 invasive lobular breast cancers with pleomorphic features which we believe is the largest series of this subtype.

## Methods

### Case selection

The records of the pathology department of Rutgers Robert Wood Johnson University Hospital were searched from 2009 to 2019 for cases of invasive pleomorphic lobular cancer. A total of 16 cases were identified with sufficient tumor volume for subsequent sequencing analysis.

The Cancer Genome Atlas was searched for breast invasive carcinoma cases harboring *CDH1* loss of function mutations or deletions with a total of 115 cases identified. Corresponding whole slide images were reviewed by an expert in breast pathology (N.B.). Twenty-two cases were diagnosed as PLC based on infiltrative pattern of classic ILC with abnormal nuclear features as has been previously described [6, 15]. An additional 9 cases were included where the outside pathology report diagnosed infiltrative or invasive pleomorphic lobular cancer or high grade (grade 3) invasive cancer while harboring *CDH1* loss.

### In-house mutation analysis

For each of the 16 cases from Rutgers Robert Wood Johnson University Hospital, 5 formalin-fixed paraffin-embedded unstained sections at 5 um were extracted using a QIAmp DNA Micro Kit (Qiagen, Hilden, Germany). DNA was sequenced using the Trusight Tumor 15 gene panel on an Illumina MiSeq instrument (Illumina, San Diego, CA).

### PLC data

Data from TCGA was accessed and analyzed using cBioPortal (http://www.cbioportal.org). Data on patient’s age, disease stage, receptor and HER2 status, and relevant genomic hotspots, copy number alterations, and fusions were obtained when available. In-house cases did not have copy number or fusion analysis performed. Data from in-house cases as well as TCGA did not include germline analysis.

## Results

### Patient and sample characteristics

Among PLC cases from TCGA, 52% were age 50–65 at diagnosis and 32% were over 65. Only 10% of these cases were stage I while 29% were stage III. The majority of cases where receptor status were known were positive for estrogen receptor (88%) and 30% were HER2 positive. The patient age and stage were more heterogeneous in PLC cases from our institution. Thirty-one percent of patients were younger than 50 and 38% were stage I at diagnosis. Similar distribution in estrogen receptor expression to TCGA were observed with 81% estrogen receptor positive while 25% were HER2 positive (Supplementary Table 1). All of the patients, both from TCGA and our institution, were female.

Overall, 79% of the patients in our study were 50 years or older at diagnosis and 34% of these were older than 65. The PLC stage at diagnosis in our study tended to be advanced with 52% stage II and 30% stage III, while 85% were positive for estrogen receptor (Table 1).

**Table 1:** Clinicopathologic and biomarker status of 47 cases of PLC

Age		Tumor		Node		Stage		Receptor status	
< 50	10	< 2 cm	11	N0	26	Stage I	8	ER+	34
50–65	21	2–5 cm	22	N1	8	Stage II	23	ER−	6
> 65	16	> 5 cm	11	N2	3	Stage III	13	PR+	31
		Unknown	3	N3	7	Unknown	3	PR−	9
				Unknown	3			HER2+	11
								HER2−	28
								Unknown	7

### Molecular alterations

Of the 31 cases identified as invasive lobular breast cancers with pleomorphic features from TCGA, 24 (77%) harbored inactivating mutations in *CDH1* (3 splice site, 5 nonsense, and 16 frameshift mutations). All but one of these showed loss of heterozygosity (LOH), and the case with an inactivating mutation without LOH had 16% *CDH1* mRNA expression. Four cases had missense mutations in *CDH1*, and all of these had 37% or less *CDH1* mRNA expression. The final 3 cases showed homozygous deletion of the *CDH1* gene locus (Table 2).

**Table 2:** Molecular alterations in 31 cases of PLC from TCGA

ID	ER IHC	PR IHC	HER2	Gene	Alteration	Gene	Alteration	Gene	Alteration	Gene	Alteration	Other alterations
TCGA-AR-A2LJ	70–79%	70–75%	pos	CDH1	E165Q	ERBB2	AMP	PIK3CA	H1047R			
TCGA-A2-A0SY	80–89%	40–49%	pos fish	CDH1	S18fs	ERBB2	AMP	PIK3CA	E545K			
TCGA-D8-A27G	> 75%	> 75%	2 ihc fish neg	CDH1	E167fs	ERBB2	AMP/ I767M	PIK3CA	H1047R			
TCGA-A8-A0A7	neg	neg	pos	CDH1	E243K	ERBB2	AMP	PIK3CA	E726K			
TCGA-A8-A0A6	pos	pos	neg	CDH1	T522_splice	ERBB2	L755S	PIK3CA	G118D			
TCGA-A8-A0AB	?	?	?	CDH1	D400fs	ERBB2	L755S	PIK3CA				BRCA2 deep del
TCGA-C8-A274	?	?	?	CDH1	P625fs	ERBB2	I767M	PIK3CA	H1047R			
TCGA-AC-A3YI	90–99%	20–29%	neg	CDH1	E763fs	ERBB2	L755S	PIK3CA				FGFR1 amp
TCGA-A2-A0T6	90–99%	90–99%	neg	CDH1	Y827fs	ERBB2	L755R (L755W, L755M) & R678Q	PIK3CA				
TCGA-BH-A0C1	pos	pos	neg	CDH1	P159fs	ERBB2	V777L	PIK3CA				
TCGA-C8-A3M7	neg	neg	neg	CDH1	T340fs	ERBB2	S305C	PIK3CA	H1047R			KMT2C Q3151*
TCGA-AO-A128	neg	neg	neg	CDH1	A817V	ERBB2	V797A	PIK3CA				TP53 R342*, KMT2C Q1787Lfs*18 & E495Dfs*42
TCGA-BH-A18P	pos	neg	pos	CDH1	S36fs	ERBB2	L755S	PIK3CA				MAP3K1 P233Qfs*30
TCGA-A8-A07B	pos	pos	pos fish	CDH1	P200fs			PIK3CA	H1047R			TP53 Q165Afs*8
TCGA-E2-A1IJ	?	?	?	CDH1	V678fs			PIK3CA		PTEN	homodel	
TCGA-A2-A0EX	pos	pos	neg	CDH1	Q511*			PIK3CA	E545K			FGFR1 amp
TCGA-E2-A2P5	pos	pos	neg fish	CDH1	Q383*			PIK3CA	N345K, G914R			TP53 F341S KMT2C E648Vfs*8
TCGA-AR-A2LL	70–79%	70–79%	neg fish	CDH1	G278R			PIK3CA		PTEN	C124S	
TCGA-E9-A3X8	20–29%	20–29%	< 10% pos (negative in report)	CDH1	515_516ins*			PIK3CA				
TCGA-BH-A0E9	pos	pos	neg	CDH1	D756fs			PIK3CA		PTEN	X70_splice	
TCGA-EW-A1J5	90–99%	90–99%	neg	CDH1	F499fs			PIK3CA	E545K, E726K			
TCGA-BH-A2L8	70–79%	80–89%	neg	CDH1	R800fs			PIK3CA	G1049R	PTEN	S287*	
TCGA-BH-A18F	pos	pos	neg	CDH1	L581fs			PIK3CA	Q546K, H1047R			
TCGA-BH-A209	pos	pos	?	CDH1	R63*			PIK3CA				ZNF791-FGFR1
TCGA-A2-A3KC	90–99%	90–99%	neg fish	CDH1	T748fs			PIK3CA	H1047R			
TCGA-AN-A03Y	pos	pos	neg	CDH1	R63*			PIK3CA	H1047R			
TCGA-A8-A09W	?	?	?	CDH1	T646_splice			PIK3CA				RB1 Y606*
TCGA-EW-A3E8	?	?	?	CDH1	I722_splice			PIK3CA	E545K			
TCGA-AO-A12G	90–99%	30–39%	neg fish	CDH1	16q22.1 homodel			PIK3CA		PTEN	R47fs	TP53 K139_P142del
TCGA-E2-A1LG	?	?	?	CDH1	16q22.1 homodel			PIK3CA				TP53 I255del KMT2c W1002* FGFR1 amp PIK3R2 amp
TCGA-A2-A25D	?	?	?	CDH1	16q22.1 homodel			PIK3CA	E110del			

Within TCGA dataset of PLC, we found 10 (32%) had activating ERBB2 mutations and 6 (19%) had ERBB2 amplification with two cases showing both amplification and an activating ERBB2 mutation. In total, 45% had either ERBB2 amplification and/or activating mutation including 7 cases where the receptor status was unknown. Among the ERBB2 activating mutations, the predominant hotspot was at codon 755 with 50% occurring at this location. The p.L755S missense mutation was observed in 4 of the 5 mutations at this position with one mutation being a p.L755R. Curiously, the TCGA annotation appears to have misclassified this alteration as two separate missense mutations, p.L755W and p.L755M, at the same variant allele frequency when in fact this is a dinucleotide substitution resulting in a single amino acid change. The remainder of the ERBB2 mutations identified also occurred in the protein kinase domain, with the exception of p.S305C which occurs in the extracellular domain.

Additionally, we queried TCGA data set for PIK3CA and PTEN alterations. Seventeen of 31 (55%) had hotspot mutations in PIK3CA with p.H1047R being the most common of these being found in 8 cases (47% of PIK3CA mutations). The next most frequent PIK3CA alteration was p.E545K in 4 cases (24%). Three cases had 2 separate hotspot mutations in PIK3CA, which recent data suggests may be particularly sensitive to inhibition [16]. We correlated PIK3CA mutations with receptor status and found that 8 cases (47%) were ER/PR positive and HER2 negative, 4 cases were ER/PR/HER2 positive (24%), 1 case was ER/PR negative and HER2 positive, 1 case was ER/PR/HER2 negative, and for three cases the receptor status was unknown. Five cases harbored PTEN alterations and in only 1 of these cases was there a coexistent PIK3CA hotspot mutation. Three of the PTEN alterations were predicted to be inactivating (1 splice site, 1 non-sense, and 1 frameshift mutation), 1 case showed homozygous deletion of the PTEN locus, and 1 case had the well-characterized catalytically dead PTEN p.C124S that completely ablates PTEN phosphatase activity [17].

Interestingly, classic ILC in TCGA had a similar percentage overall percentage PI3K pathway alterations compared with PLC, with 38/84 cases having an activating PIK3CA alteration and 9/84 a PTEN alteration with one case showing alterations in both PIK3CA and PTEN compared. However, there were no activating ERBB2 mutations and only one case with ERBB2 amplification (1%) in the classic ILC in TCGA (Supplementary Table 2).

In total, 27 out of the 31 cases (87%) from TCGA had what would be considered a driver mutation in ERBB2 or the PIK3CA/PTEN pathway. In further examining these 4 cases without these alterations, the results for HER2 FISH or IHC were unknown for 3 (although TCGA sequencing did not report amplification of ERBB2). One of these cases had a ZNF791-FGFR1 fusion while another case had FGFR1 and PIK3R2 amplifications.

Finally, we cataloged TP53 mutations in PLC TCGA cases and found this gene altered in 5 out of 31 cases (2 in-frame deletions in the DNA binding domain, 1 frameshift mutation in the DNA binding domain, 1 missense mutation in the tetramerization domain, and 1 truncating mutation in the tetramerization domain).

Within our in-house cases of PLC, we found 3 out of 16 (19%) had activating ERBB2 mutations and 4 (25%) had ERBB2 amplification with two cases showing both amplification and an activating ERBB2 mutation. In total, 5 (31%) had either ERBB2 amplification and/or activating mutation. Among the ERBB2 activating mutations, the predominant hotspot was again the p.L755S missense mutation which was seen in 2 of the 3 cases. The other ERBB2 mutation identified, p.E770_A771insAYVM, also occurred in the protein kinase domain (Table 3).

**Table 3:** Molecular alterations in 16 cases of PLC from our institution

ID	ER IHC	PR IHC	HER2	Gene	Alteration	Gene	Alteration
1	3+	0	1+ neg	PIK3CA	H1047L		
2	99% 3+	50% 2–3+	neg	Not detected			
3	0	0	pos fish	ERBB2	L755S	TP53	S15Rfs*18
4	0	0	neg	ERBB2	L755S	PIK3CA	P539R
5	99% 3+	99% 3+	neg	Not detected			
6	95% 3+	70% 3+	neg	PIK3CA	H1047R		
7	95% 3+	90% 3+	neg	Not detected			
8	99% 3+	99% 3+	neg	Not detected			
9	100% 3+	60% 3+	neg	AKT1	E17K		
10	95% 3+	95% 3+	neg	PIK3CA	H1047R		
11	95% 3+	80% 3+	neg	Not detected			
12	95% 3+	90% 3+	neg	Not detected			
13	95% 3+	0	pos	ERBB2	E770_A771insAYVM		
14	0	0	pos	PIK3CA	Q546E	TP53	R248Q
15	95% 2–3+	95% 2–3+	neg	PIK3CA	E545K	TP53	G108S
16	95% 3+	80% 3+	pos	TP53	R175H		

**Fig. 1:**
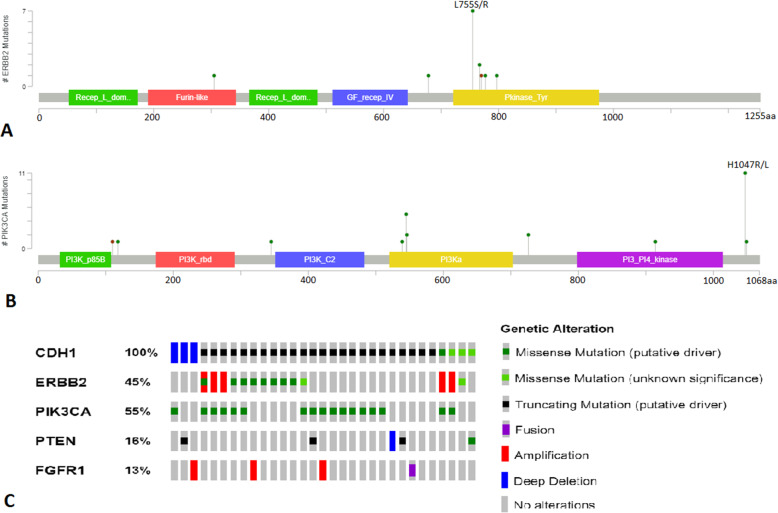
The distribution of **a** ERBB2 and **b** PIK3CA mutations observed in our study. **c** The distribution and types of ERBB2, PIK3CA, PTEN, and FGFR1 alterations in PLC cases from TCGA

While the next-generation sequencing panel used for the in-house did not include PTEN, it did cover AKT1 in addition to PIK3CA. Six of 16 cases (38%) had PIK3CA hotspot mutations with codon 1047 again the most common (2 p.H1047R and 1 p.H1047L) (Fig. 1). In addition, 1 case was found to have the AKT1 p.E17K mutation. In correlating PIK3CA hotspot mutations with receptor status, we found that 3 cases were ER/PR positive and HER2 negative, 1 case was ER positive and PR/HER2 negative, 1 case was ER/PR negative and HER2 positive, and 1 case was ER/PR/HER2 negative.

Within the in-house PLC cases, 10/16 cases (63%) had what would be considered a driver mutation in ERBB2 or the PIK3CA/PTEN pathway.

We identified TP53 mutations in 4/16 cases (25%) with 3 being missense mutations in the DNA binding domain and 1 frameshift mutation at codon 15.

## Discussion

In the current study, PLC cases were associated with older age while the stage at diagnosis was more advanced compared to breast cancer not otherwise specified with 52% stage II and 30% stage III at diagnosis in agreement with previous reports [18] (Table 1).

An important observation from our study of 47 PLC cases was the 28% had *ERBB2* mutations, considerably higher than the 5% previously reported in ILC not otherwise specified [1]. In a study of 24 PLC or pleomorphic lobular carcinoma in situ, Lien and colleagues reported an ERBB2 mutation frequency of 20.8% of invasive or in situ pleomorphic carcinomas compared to only 2% of classic ILC [12]. However, they note all of their patients were Taiwanese and it is unclear whether *ERBB2* mutation frequencies differ among ethnicities. Similarly, a recent study from Rosa-Rosa et al. reported 26% *ERBB2* mutations in 27 PLC patients in Spain [14]. Finally, a study from Massachusetts of 17 patients with PLC found 3 (18%) with *ERBB2* mutations [13]. Our results add credence that ERBB2 mutations are prevalent among PLC patients. This is clinically important as case reports have shown that patients with ERBB2-mutated breast cancers respond to targeted HER2 targeted therapy [19, 20]. This was confirmed by responses to neratinib seen in ERBB2-mutated caners in the SUMMIT trial [21]. Additionally, the majority of HER2 mutations in breast cancer are reported to be activating and respond to HER2 inhibition [22]. One caveat in treating these HER2 mutant breast cancers is a recent report suggesting dual targeting of HER2 and ER pathways may be required for optimal treatment in cases that are also positive for estrogen receptor [23]. There is also a clear need to investigate role of adjuvant HER2 inhibitors in early stage HER2 mutant cancers.

In addition to ERBB2 activating mutations, we found that 21% of PLC cases in our study had ERBB2 amplification, considerably higher than the 2–4% ERBB2 overexpression reported in classical or ILC not otherwise specified [14, 24]. The overall percentage of ERBB2 amplification was similar to that reported by Lien of 33% in invasive or in situ pleomorphic carcinomas. ERBB2 amplification and mutation were not completely mutually exclusive in our study. We found 4 cases with co-occurring ERBB2 amplification and mutation such that the overall percentage with an ERBB2 alteration was 43%, including 8 cases from TCGA where ERBB2 IHC or FISH were unknown but sequencing did not show evidence of copy number gain.

Another important finding from our study was that 49% of PLC cases had activating PIK3CA mutations. While this frequency is similar to the reported frequency of 53% PIK3CA mutations by Zhu and colleagues [13], we believe this is the first study of PLC that examines the overall percentage of cases which had an alteration in ERBB2 or the PI3K pathway. Within PLC cases from TCGA, we found that 87% had an alteration in ERBB2, PIK3CA, or PTEN and 74% had an alteration in ERBB2 or PIK3CA. This is highly relevant with the introduction of alpha-specific PI3K inhibitors clinically [25]. Specifically, the US Food and Drug Administration has granted approval to alpelisib in combination with fulvestrant for hormone receptor-positive, HER2-negative, PIK3CA-mutated advanced, or metastatic breast cancer following on endocrine therapy based on the SOLAR-1 trial [26]. There is conflicting evidence as to whether PTEN deficiency may be targeted with alpha-specific PI3K inhibitors. Alpelisib has been reported to effectively inhibit growth in lipoma cells deficient in PTEN derived from PTEN hamartoma tumor syndrome patients, but loss of function PTEN mutations has also been reported as a mechanism of resistance to alpelisib and fulvestrant in PIK3CA mutant hormone receptor-positive breast cancer [27, 28]. Among the 4 cases of PLC from TCGA which did not harbor alterations in ERBB2, PIK3CA, and PTEN, we found that two of the cases had alterations in FGFR1, an FGFR1 amplification and a ZNF791-FGFR1 fusion, suggesting the potentially targetable alterations within this group may be 94% (Fig. 1c). A case report has shown activity in FGFR1-amplified breast cancer with the FGFR inhibitor pazopanib and erdafitinib has shown activity in FGFR-altered urothelial carcinoma [29, 30].

## Conclusions

Our results show that PLC presents at more advanced stage than breast cancer not otherwise specified and harbors high percentages of both ERBB2 alterations and PI3K pathway alterations. Due to the fact that PLC tends to be more aggressive, the finding that high percentages contain alterations which can be targeted therapeutically is clinically relevant and suggests testing for these alterations is warranted in these cases. As clinical sequencing of cancer becomes more routine, this is technically feasible, but the role of sequencing and adjuvant therapy in early stage cancers, including PLC, merits further investigation.

## Supplementary Information

**Additional file 1: Supplementary Table 1.** Clinicopathologic and biomarker status in cases of PLC from TCGA and our institution.

**Additional file 2: Supplementary Table 2.** Molecular alterations in 84 cases of classic ILC from TCGA.

## Data Availability

All data generated and analyzed during this study are included in this published article [and its supplementary information files].

## Abbreviations

IDC: Invasive ductal carcinoma
ILC: Classic invasive lobular cancer
LOH: Loss of heterozygosity
PLC: Invasive pleomorphic lobular carcinoma of the breast
TCGA: The Cancer Genome Atlas
